# Spatial Patterns of Ectomycorrhizal Assemblages in a Monospecific Forest in Relation to Host Tree Genotype

**DOI:** 10.3389/fpls.2013.00103

**Published:** 2013-04-24

**Authors:** Christa Lang, Reiner Finkeldey, Andrea Polle

**Affiliations:** ^1^Forest Botany and Tree Physiology, Büsgen-Institut, Georg-August Universität GöttingenGöttingen, Germany; ^2^Forest Genetics and Forest Tree Breeding, Büsgen-Institut, Georg-August Universität GöttingenGöttingen, Germany

**Keywords:** belowground interactions, community ecology, ectomycorrhiza, deciduous forest, intraspecific variation, interspecific variation

## Abstract

Ectomycorrhizas (EcM) are important for soil exploration and thereby may shape belowground interactions of roots. We investigated the composition and spatial structures of EcM assemblages in relation to host genotype in an old-growth, monospecific beech (*Fagus sylvatica*) forest. We hypothesized that neighboring roots of different beech individuals are colonized by similar EcM assemblages if host genotype had no influence on the fungal colonization and that the similarity would decrease with increasing distance of the sampling points. The alternative was that the EcM species showed preferences for distinct beech genotypes resulting in intraspecific variation of EcM-host assemblages. EcM species identities, abundance and exploration type as well as the genotypes of the colonized roots were determined in each sampling unit of a 1 L soil core (*r* = 0.04 m, depth 0.2 m). The Morisita-Horn similarity indices (MHSI) based on EcM species abundance and multiple community comparisons were calculated. No pronounced variation of MHSI with increasing distances of the sampling points within a plot was found, but variations between plots. Very high similarities and no between plot variation were found for MHSI based on EcM exploration types suggesting homogenous soil foraging in this ecosystem. The EcM community on different root genotypes in the same soil core exhibited high similarity, whereas the EcM communities on the root of the same tree genotype in different soil cores were significantly dissimilar. This finding suggests that spatial structuring of EcM assemblages occurs within the root system of an individual. This may constitute a novel, yet unknown mechanism ensuring colonization by a diverse EcM community of the roots of a given host individual.

## Introduction

In Central Europe, beech (*Fagus sylvatica*) is a dominant, ecologically, and economically important tree species (Ellenberg and Strutt, 2009). In mono- and hetero-specific forests roots compete for limited resources of water and nutrients (Bobowski et al., 1999; Jackson et al., 1999; Linder et al., 2000; Brunner et al., 2001; Hölscher et al., 2002; Meinen et al., 2009a,b). In mixtures beech roots were often the superior competitor compared with other tree species (Schmid and Kazda, 2002; Bolte and Villanueva, 2006; Rewald and Leuschner, 2009). With the advent of molecular techniques, genotyping of tree individuals of the same species became possible and was applied to study the intraspecific patterns of root soil occupation (Brunner et al., 2004; Lang et al., 2010). Genotyping of beech roots revealed no evidence for competition of tree individuals for soil exploration (Lang et al., 2010).

However, nutrient uptake by beech roots is primarily achieved by ectomycorrhizal (EcM) fungi, which colonize the root tip and form a new compound organ, the EcM. EcM enwrap the root tip by a mantle-like structure from which hyphae emanate into the soil. Thereby, EcM enlarge the surface for soil exploration and can overcome nutrient depletion zones (Cairney, 2011). Beech trees form EcM with a large number of different fungal species (Buée et al., 2005; Pena et al., 2010; Lang et al., 2011). Functional traits for nutrient acquisition vary among different EcM species including biochemical and morphological features such as exudation of organic acids for nutrient solubilization, exudation of hydrolytic and oxidative enzymes as well as different hyphal lengths which enable different EcM to forage in different soil volumes (Courty et al., 2010; McGuire et al., 2010; Plassard et al., 2011; Pritsch and Garbaye, 2011; Agerer et al., 2012; Weigt et al., 2012; Pena et al., 2013). If different EcM species provided different benefits, we expect that neutral behavior for resource competition in mono-specific beech forests is mediated by mixed EM fungal assemblages with no preference for individual trees.

However, there is now evidence that the ability for mycorrhization with distinct fungal species is under genetic control of the host (Peterson and Bradbury, 1998). For example, greenhouse studies with Scots pines from different seed sources and with different Norway spruces clones showed strong intraspecific host differences in colonization and EcM species composition (Leski et al., 2010; Velmala et al., 2012). Mycorrhizal colonization of poplar hybrids and their parents varied strongly and affected EcM enzymatic activities suggesting a genetic basis for plant-EcM interactions (Tagu et al., 2001; Courty et al., 2011). Furthermore, in a poplar plantation differences in EcM community composition were found among different transgenic poplars modified in lignification enzymes and also among different *P*. x *euramericana* clones (Danielsen et al., 2013). Because of the significance of EcM for plant nutrition and ecosystem functioning, it is important to understand the links between inter- and intraspecific plant and mycorrhizal diversity.

The aim of our study was to investigate the relationship between EcM fungal assemblages and the roots of individual beech trees. Since beech propagates typically by seedlings, each tree is usually a distinct genotype. In mono-specific forests roots of a given individual are strongly intermingled with those of the neighboring trees, even close to the stem the individual (Lang et al., 2010). Therefore, analyses of the relationship between roots of distinct trees and their mycorrhizal assemblage require root genotyping and fungal species identification of that specific root. We used this strategy to test our working hypothesis that the EcM species composition of different neighboring root genotypes is more similar than that of the same genotype sampled at different positions. The alternative was that the EcM species showed preferences for distinct beech genotypes resulting in intraspecific variation of host fungal assemblages. For the purpose of this study we defined the roots in our sampling unit of a 1L-soil core (*r* = 0.04 m, depth 0.2 m) as neighboring roots (small spatial scale) compared with roots in different soil cores collected at distances of 1–9 m within a plot and those collected in different plots at distances of about 40 m. We analyzed the EcM species abundances and identities on all root tips in each soil core and determined the genotypes of colonized roots. We used these analyses to describe the spatial pattern of EcM diversity and to investigate the similarities of EcM assemblages on roots of different beech genotypes at small and larger spatial scales.

## Materials and Methods

### Study site and sampling

**Figure 1:**
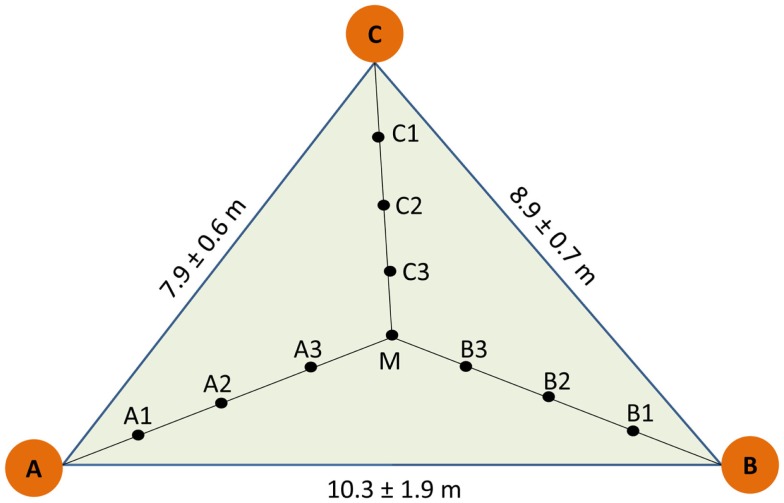
**Scheme of sampling design**. A, B, and C indicate the position of the target trees used to construct the sampling triangle. Three plots were selected and distances indicate mean of the three plots. Soil cores were collected at equidistant positions within the triangle (A1A2 = A2A3 = A3M = 1.4 ± 0.2 m, B1B2 = B2B3 = B3M = 1.3 ± 0.3 m, C1C2 = C2C3 = C3M = 1.2 ± 0.1 m).

The study was conducted in the area of the National Park Hainich (Thuringa, Germany, 51°05′28′′N, 10°31′24′′E), where the long-term annual sum of precipitation is 670 mm and the annual mean temperature 7.5°C (Leuschner et al., 2009). The soil type is Stagnic Luvisol developed from loess on limestone with an acidic pH 5.1, C/N ratio of about 30 in the humus layer and an organic carbon content of 2.9–3.7 kg m^−2^ (Guckland et al., 2009). An area of 100 × 100 m was selected in a long-term unmanaged, old-growth beech stand in the northeastern part of the National Park, in which three plots at distances of about 40 m were set up as described before (Lang et al., 2010). Each plot consisted of three trees with a mean stem diameter of 51 ± 5 cm, denominated as A, B, and C respectively. The trees formed a triangle (Figure 1). For sampling the geometric center (M) was determined and three further sampling points were determined at regular distances between M and the stem each of the trees (Figure 1). This design resulted in 10 sampling points per triangle and 30 samples in total. Soil cores of a volume of 1 L (radius of 0.04 m, depth of 0.2 m) were collected in June 2009. Triangle-forming trees A, B, and C and their neighbors were mapped and leaves were collected for microsatellite analyses (Lang et al., 2010).

### Analyses of roots and ectomycorrhizas

The roots were removed from the soil core by careful washing and stored at 4°C between moist tissue papers. All root fragments of each soil core were used for mycorrhizal analysis. For this purpose the roots were spread under a compound microscope (Leica M205 FA, Wetzlar, Germany) and all roots tips were counted and classified as either dead, vital non-mycorrhizal, or vital mycorrhizal root tips. The vital mycorrhizal root tips were morphotyped after a simplified method of Agerer (1987/2006) using color, texture of the EcM mantel, branching, abundance of external hyphae, and rhizomorphs as classification criteria. Exploration types were assigned after Agerer (2001), Courty et al. (2008, Supplementary material S1) and Pena et al. (2010). Pictures were taken and deposited together with the fungal description and molecular information (see below) under http://www.uni-goettingen.de/de/92389.html. The abundance of each EcM morphotype on each root fragment was recorded. Aliquots of each fungal morphotype (10–20 root tips) were collected and stored at −80°C. After mycorrhizal analysis, aliquots of the root fragments were also stored (−80°C). Coarse and fine roots (<2 mm diameter) were separated and weighed.

To determine EcM species identities DNA was extracted from the morphotypes with the DNeasy Mini Plant Kit (Qiagen, Hilden, Germany). The internal transcribed spacer (ITS) region of the fungal rDNA was amplified by using the primer pair ITS5 and ITS4 (MWG, Biotech, Ebersberg, Germany) after White et al. (1990). The PCR conditions and sequencing procedures have been reported before (Pena et al., 2010). For fungal identification, BLAST searches were carried out against the NCBI^1^ and UNITE^2^ public sequence databases. Sequences were assigned matching species names when the BLAST matches showed identities higher than 97% and scores higher than 800 bits. If no appropriate match was found, the sequence was assigned a higher-level taxonomic name or was called an uncultured ectomycorrhizal fungus (UECM) and numbered. The sequences have been deposited in NCBI with the following accession numbers: EU346875, EU816604, EU816608, EU816609, EU816611, EU816616, EU816619, EU81662, EU816623, EU816625, EU816642, EU816643, EU816646, EU816647, EU816653, EU816654, EU816670, EU816679, EU826353, HQ336683, HQ336695, HQ336696, HQ336697, and HQ336701. *C. geophilum* was determined as black morphotype.

Genotyping of roots and leaves has been reported before (Lang et al., 2010). Briefly, individual trees and their roots were identified by sequence analyses of four highly polymorphic microsatellite loci (*sfc0018, sfc0161, sfc1143, sfc1063*), previously developed for *Fagus crenata* (Asuka et al., 2004) and tested for *Fagus sylvatica* (Lang et al., 2010).

### Data analysis

Mycorrhizal colonization (%) was calculated as: number of vital mycorrhizal root tips × 100/(number of vital mycorrhizal root tips + number of vital non-mycorrhizal root tips). The vitality index of the root tips was calculated as: (number of vital mycorrhizal root tips + number of vital non-mycorrhizal root tips) × 100/(number of vital mycorrhizal root tips + number of vital non-mycorrhizal root tips + dead root tips). The Shannon–Wiener index for roots of different tree genotypes in a soil core was calculated on the basis of the relative abundance of fine root biomass per individual tree. The Shannon–Wiener index for EcM species on the root tips in a soil core was calculated on the basis of the abundance of the EcM species per total number of root tips in the soil core. This yielded Shannon–Wiener indices for the diversity of individual trees present in a sample (*H*′_tree_) or for the diversity of EcM in a sample (*H*′_EM_) with *H*′ = −Σ*pi* ln *pi*, where *p* is the relative abundance of the tree genotype *i* or the relative abundance of the EcM species *i* (Shannon and Weaver, 1949).

Similarity indices were calculated as generalized Morisita–Horn index *C*_*qN* by comparing *N* communities on species information shared by at most *q* communities using the procedure developed by Chao et al. (2008) and implemented in the program SPADE by Chao and Shen (2010)^3^. EcM species abundances per soil core, per tree genotype, or per soil core and tree genotype were used as input parameters and run with a bootstrap value of 200.

Statistical analyses and curve fitting were performed with STATGRAPHICS Centurion (Statistical Graphics Corp., Warrenton, USA) or ORIGIN 7.0 (Origin Lab Corp., Northampton, USA). When the data were not-normal distributed two sample comparisons were conducted with the Mann–Whitney *W*-test for medians.

## Results

### Interspecific fungal diversity in relation to intraspecific host diversity

**Figure 2:**
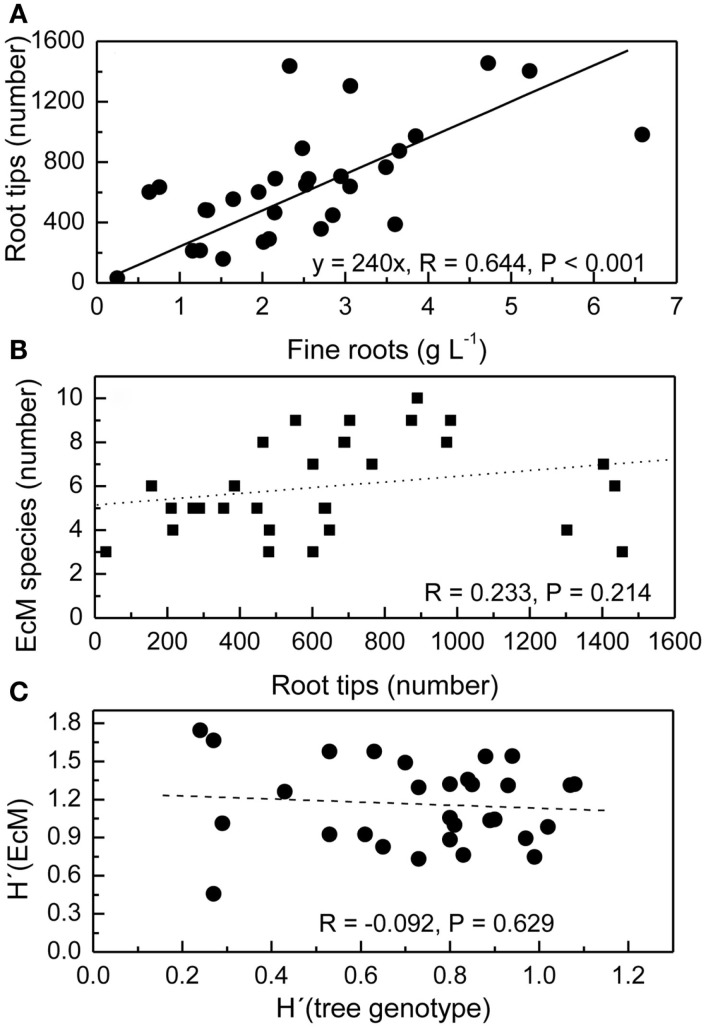
**Relationships between the number of root tips and fine root mass per soil core (A), the number of ectomycorrhizal fungal species (EcM) and the number of root tips per soil core (B), and the Shannon–Wiener index H′of EcM diversity and root genotype diversity H′ (tree genotype) per soil core (C)**. The volume of the soil core was 1 L. All root tips were counted and analyzed.

The mean fine root biomass in the top 20 cm of the soil was 2.5 ± 0.3 g L^−1^ and not affected by the distance of the soil core from the next tree (Lang et al., 2010). However, in individual soil cores the amount of fine roots was variable with increasing amounts of fine roots corresponding to increasing numbers of root tips (Figure 2A). Because the number of EcM species detected in ecosystems depends on the sampling effort (Taylor, 2002), we expected that the number of different EcM species would increase with increasing number of root tips. We found between 3 and 10 EcM species per soil core, but these numbers were not related to the number of root tips in that soil core (Figure 2B). The Shannon–Wiener diversity index of the EcM community in a soil core was neither affected by the number of root tips in that soil core (*R* = 0.021, *P* = 0.909, not shown).

We have previously reported that roots of 21 beech genotypes were identified in the three study plots with a mean of 3.3 ± 0.2 individuals per soil core and *H*′_tree genotype_ ranging from 0.27 to 1.08 (Lang et al., 2010). We plotted the interspecific fungal diversity per soil core H′_EM_ against H′_tree_ in this soil core to find out whether higher intraspecific host diversity was related to higher interspecific diversity of the EcM fungi on the roots (Figure 2C). No significant correlation was observed (Figure 2C).

### Spatial patterns of EcM species richness and abundances in the study plots

**Figure 3:**
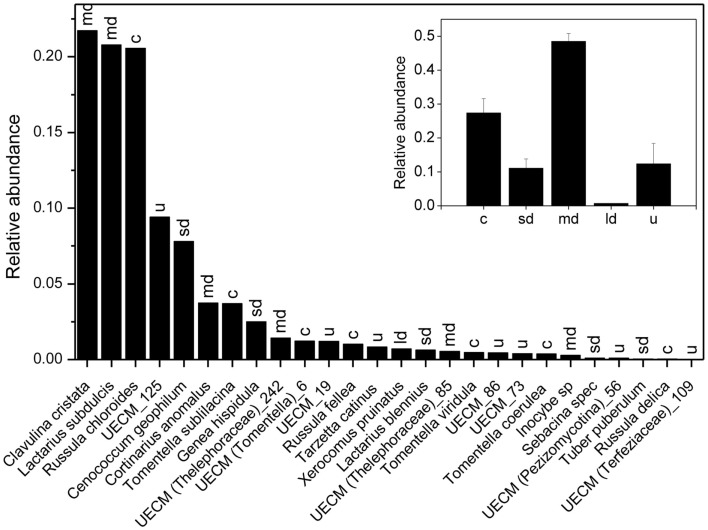
**Relative abundance of ectomycorrhizal fungal species on root tips of beech (*Fagus sylvatica*)**. The sum of all EcM root tips of the three plots analyzed was set as 1. Letters above bars indicate exploration types: c = contact, sd = short distance, md = medium distance, ld = long distance, u = unknown. The inset shows the relative contribution of different EcM exploration types to root tip colonization.

We recorded 6801, 7248, and 5578 vital mycorrhizal root tips on plot 1, 2, and 3, respectively. Mycorrhizal root tip colonization (99.3 ± 1.4%, *P* = 0.48) and root vitality (32.4 ± 1.6%, *P* = 0.31) did not differ between the three plots. We found a total number of 26 EcM species, of which 8 colonized together 90% of the mycorrhizal root tips (Figure 3). *Clavulina christata, Russula chloroides*, and *Laccaria subdulcis* were the most abundant species, followed by UECM-125, *Cenococcum geophilum*, *Tomentella sublilacina*, *Cortinarius anomalus*, *Genea hispidula* (Figure 3). Analysis of fungal exploration types revealed that about 50% of the root tips were colonized with medium distance fungi and 30% with contact types, whereas short and long distance exploration type fungi colonized only about 10% of the root tips (Figure 3, inset).

**Figure 4:**
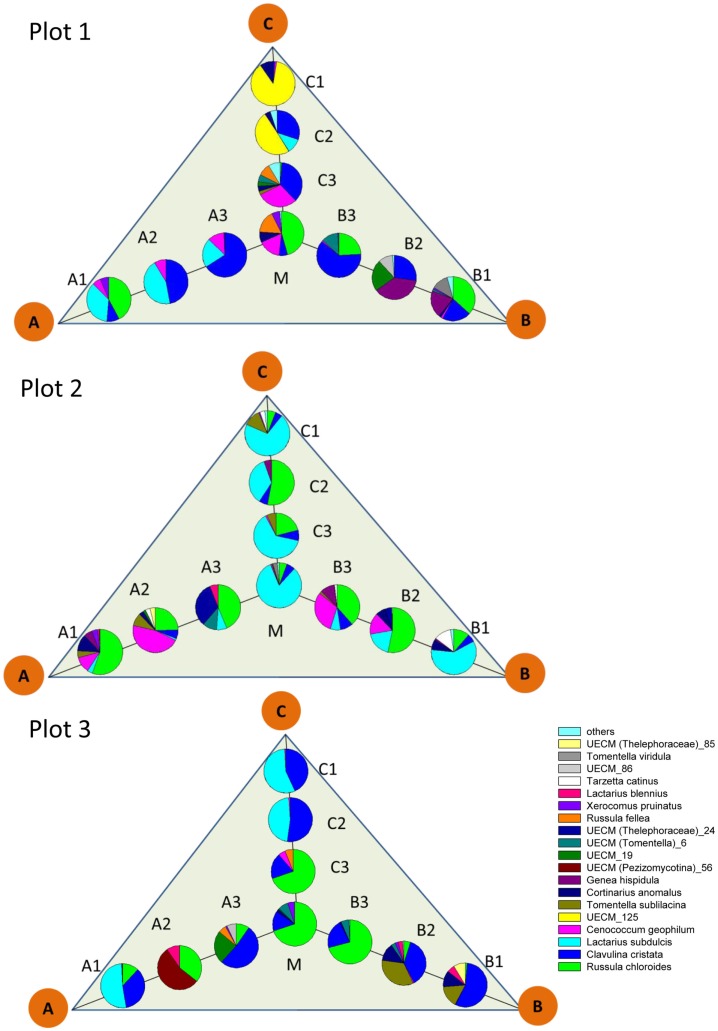
**Scheme of localization and relative abundance of EcM species in 1 L soil cores**. Abbreviations for the positon of the soil cores as in Figure 1. Others: sum of UECM_73, *T. coerulea*, *Inocybe* sp., *Sebacina* sp., *T. puberulum*, *R. delicia*, UECM (Terfeziaceae)_109, which accounted together for <1% of the total EcM abundance.

The pattern of EcM species abundance in different soil cores revealed large variations in the fungal assemblages (Figure 4, Table 1). While the roots in some soil cores were strongly dominated by one or two fungi, others contained higher species richness (Figure 4). To investigate the similarity between EcM fungi in different soil cores, we used the Morisita–Horn index, which is based on the relative abundance of species, by multiple community comparisons as introduced by Chao et al. (2008). Analysis of the fungal patterns for all sample combinations in a plot revealed that the similarity indices covered the whole range from almost zero (no overlap of EcM) to almost 1 (complete overlap of EcM, Table 1). The mean similarity of all plots was moderate and significantly lower between plot 1 and 2 than in the other combinations (Table 2). The similarities between the plots were much higher when the EcM were categorized after exploration types than after species (Table 2).

**Table 1:** **Morisita–Horn similarity indices for the EcM species composition in all combinations of soil cores within a plot (based on shared information between any two communities)**.

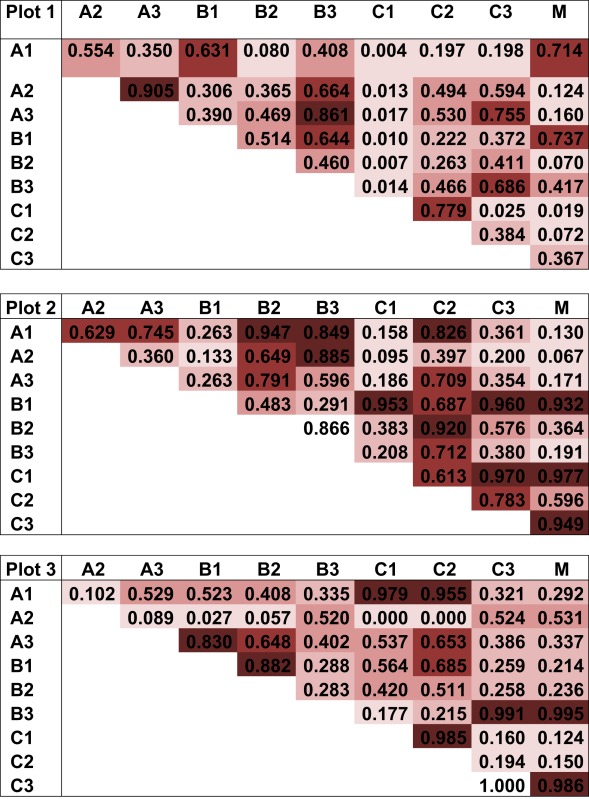

*Analyses were based on EcM species abundance. Increasingly intense colors indicate increasing similarity. Species are shown in Figure 4*.

**Table 2:** **Morisita–Horn similarity indices for the comparison of all three plots (based on shared information between any two communities) and for the three plots among each other**.

Plot	Level	Similarity index ± SE	95% Confidence interval
All	Species	0.586 ± 0.007	(0.573, 0.599)
1_2	Species	0.392 ± 0.010	(0.373, 0.412)
1_3	Species	0.689 ± 0.009	(0.671, 0.707)
2_3	Species	0.660 ± 0.011	(0.638, 0.682)
All	Ex type	0.832 ± 0.006	(0.821, 0.843)
1_2	Ex type	0.791 ± 0.008	(0.775, 0.808)
1_3	Ex type	0.770 ± 0.008	(0.754, 0.786)
2_3	Ex type	0.921 ± 0.006	(0.910, 0.933)

*Analyses were based on species abundance (species) or on exploration type (ex type). SE, standard error*.

**Figure 5:**
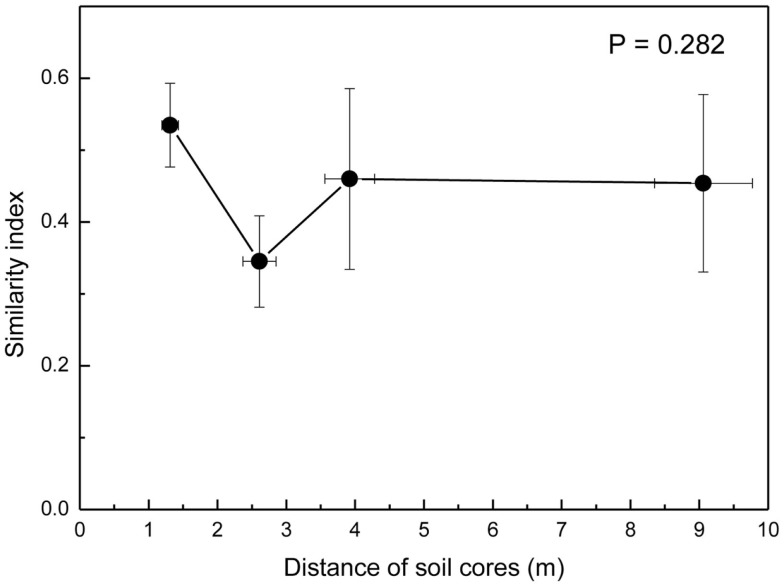
**Morisita–Horn similarity indices of ectomycorrhizal assemblages calculated for pairs of soil cores at increasing distance from each other**. 1.3 m = neighboring cores, 2.6 m = every second core (e.g., A1_A3, A2_M, B1_B3, etc), 3.8 m = every third core (e.g., A1_M, B1_M, etc), and 9 m = largest distances within plots (A1_B1, A1_C1, B1_C1). Data indicate means for the three plots.

Because the hyphae of EcM fungi can grow several meters and generate large belowground networks connecting trees (Beiler et al., 2010), we compared the overlap of fungal communities by Morisita-Horn similarity indices (MHSI) of EcM assemblages of neighboring soil cores with those of increasing distance. EcM communities in neighboring soil cores (mean distance 1.3 m) were slightly more similar than in cores collected at distances of about 2.6 m, but overall there were no significant differences up the largest distances between the positions of soil cores within a plot (Figure 5). This shows that the similarity of EcM assemblages did not decrease with increasing distances as one might have expected.

### Fungal assemblages in relation to host genotype

**Figure 6:**
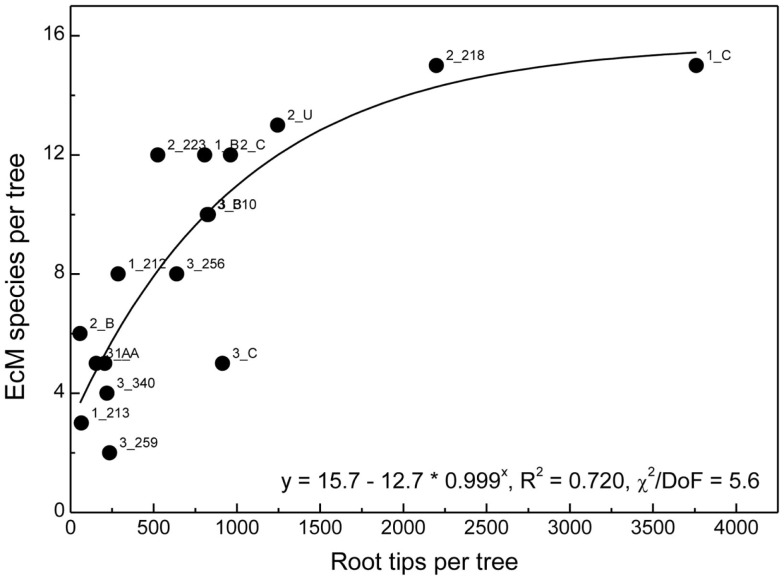
**Ectomycorrhizal species richness on roots of a distinct beech genotype in relation to the number of root tips found for this individual in all samples**. Labels indicate plot number followed by tree number. The position of all trees in the plots has been shown in Lang et al. (2010). The data were fitted by a Boltzman function.

We combined all roots found for a distinct beech genotype and determined EcM species richness per host genotype. As the number of root tips per host genotype was highly variable, we analyzed the relationship between the number of detected root tips and EcM species richness (Figure 6). A saturation curve was found suggesting that many trees were undersampled and that therefore the comparison of fungal assemblages between all different genotypes would have been biased by differences in sample abundance.

**Figure 7:**
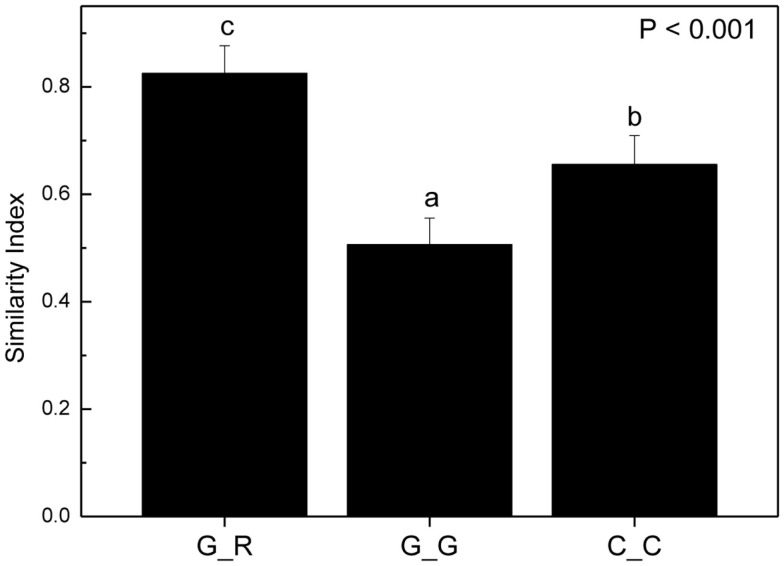
**Morisita–Horn similarity indices for combinations of ectomycorrhizal species assemblages on the roots of a distinct beech genotype with those on roots of other beech genotypes in the same soil core (G_R, *n* = 29), with the same beech genotype in different soil cores (G_G, *n* = 31) and of the EcM assemblages in all soil cores used for this analysis (C_C, *n* = 26)**. Bars indicate means ± SE. Different letters indicate significant differences at *p* < 0.05.

To circumvent this problem, we reasoned that if there were preferences of EcM species for distinct beech individuals, the EcM assemblages on roots of a given genotype should be more similar to those of the same genotype in other soil cores than to the EcM communities of other host genotypes in same soil core. To test this hypothesis we identified trees whose roots were found in two or more soil cores and used only those soil cores which contained also a reasonable number of root tips of other beech genotypes as well (means per sample: 235 ± 70). According to these criteria we identified the following trees on plot 1: A, B, C, on plot 2: 223, 218, C, U1 and on plot 3: B, 310, and 256 (cf. Figure 6). We calculated similarity indices for the EcM assemblages of a given genotype in different soil cores (G_G) and for the given genotype with the EcM community of the roots of the other genotypes in the same soil core (G_R, Figure 7). The similarity of the EcM communities in the same soil core was very high regardless of the genotype (G_R), whereas the EcM on the same beech genotype in two adjacent soil cores (G_G) were dissimilar (Figure 7). The similarity of the total EcM communities in the soil cores (C_C) used for this analysis was intermediate between G_R and G_G (Figure 7) and similar to the mean Morisita–Horn index found for the three plots (Table 2).

## Discussion

In recent years considerable efforts have been made to describe and interpret the ecological significance of spatial patterns of EcM (O’Hanlon, 2012). A key challenge is to find out whether predictable relationships exist between inter- and intraspecific plant and mycorrhizal fungal diversity, which may be key factors in understanding ecosystem functioning (Johnson et al., 2012). The present study contributes to this question by linking EcM species patterns to beech genotypes with a spatial resolution of about 0.04–9 m. The fungal community composition on the beech roots of our study and their general structures with few dominant and many scarce species are typical for Fagaceae forests (Buée et al., 2005; Courty et al., 2005; Lang and Polle, 2011; Lang et al., 2011). Because we found almost complete colonization of vital root tips with EcM, all nutrients taken up by a beech tree must have passed the EcM. Therefore, EcM are expected to play an important role in the distribution of nutrients between conspecific neighbor trees and may lead to asymmetric competition, if the EcM species differed in functions and preferences for distinct genotypes.

Data regarding functional classifications of EcM are still incomplete. EcM fungi exude different exoenzymes to mobilize nutrient resources (Cairney, 2011; Plassard et al., 2011; Pritsch and Garbaye, 2011; Hobbie and Högberg, 2012; Habib et al., 2013), but groupings for these traits are still lacking because of strong variations of the enzyme activities with their biotic and abiotic environment (Courty et al., 2008). Currently, the most frequently used classification system assigns EcM fungi according to their hyphal morphology such as lengths, densities, and surface properties to different soil exploration types, which reflect spatial differences for nutrient absorption (Agerer, 2001). In our study the abundant EcM include contact (*R. chloroides, T. sublilacina*), short distance (*C. geophilum, Genea hispidula*) and medium distance (*C. christata, L. subdulcis, C. anomalus*) soil exploration types, with a potential reach of up to 16 cm per cm EcM length (Agerer, 2001; Agerer et al., 2012; Weigt et al., 2012). Thus, the majority of EcM species can forage for nutrients beyond the dimensions of the soil core. A yet larger outreach is achieved by long distance rhizomorph-forming fungi with an exploration potential >400 cm per cm of EcM length (Agerer et al., 2012), which colonized, however, only a small fraction of the root tips in our study (about 1%, *X. pruinatus*). In other forest communities the abundance of rhizomorphic exploration types was found to be very high (Heinonsalo et al., 2007). Here, the similarity of EcM species among the plots used in our study was only moderate, but the similarity based on exploration types was very high. This finding suggests that there were no major differences between the plots with respect to soil foraging by EcM.

Previous studies in a pine forest have shown that EcM communities were highly similar at scales <3.4 m (Pickles et al., 2012). In our study we also found high similarities of EcM communities within the plots, but no significant differences between adjacent (ca. 1 m) and more distant (ca. 9 m) sampling points. Fine scale analyses of EcM at the cm-scale showed that some fungi, e.g., *Clavulina* sp. and *Cortinarius* sp., which were also present in our study, can form mycelial and EcM patches, whereas this was not the case for *C. geophilium* (Genney et al., 2006; Pickles et al., 2010). Clusters for *Cortinarius* and other fungal species (*Tomentella, Piloderma*) were also detected on oak (Gebhardt et al., 2009). The formation of clusters indicates non-random spatial structuring of the EcM communities. It has been suggested that interspecific competition or priority effects could lead to spatial partitioning of fungal species on the root system (Pickles et al., 2012).

Another possibility, which was addressed in our study, is that intraspecific host diversity may lead to structuring of the fungal assemblages. Since strong host preferences of EcM species have been found in mixtures of beech with other deciduous tree species (Lang et al., 2011) and effects of the host genotype were reported under experimental conditions (Tagu et al., 2001; Leski et al., 2010; Courty et al., 2011; Danielsen et al., 2013), it is clear that links exist between the fungal assemblage and the host genotype. However, in the present investigation we found no evidence for discernible EcM communities on distinct beech genotypes. One reason may be that the genetic structure of the genotypes studied in this old-growth unmanaged stand might have been relatively similar because the trees were established by natural regeneration and significant family structures were found in the plot (Rajendra, 2011). To further address the question of interactions between host genotype and fungal assemblages, field studies with different beech ecotypes/populations will be required.

The most striking finding of our study was that the similarity of EcM communities of different beech genotypes within a soil core was almost twice higher than for same genotype in different soil cores. Because the dimensions of the soil core were smaller than the radius of most fungal hyphae, it is possible that the same fungal genotype colonized neighboring roots of different host trees in the same soil core. Although we have not determined fungal genets, this assumption is not unreasonable because others have shown that fungal genets connect hetero- as well as conspecific neighbors (Curlevski et al., 2009; Beiler et al., 2010). The connectivity was especially strong for old, dominant individuals, where one individual was connected with as many as more than 40 other conspecific trees and could cover distances of up to 20 m (Beiler et al., 2010). Mycorrhizal networks may facilitate resource transfer within the fungal web and between, thereby, foster the establishment of seedlings with access to the common mycorrhizal network (Teste and Simard, 2008).

In our study the high dissimilarity of fungal assemblages at roots of the same genotypes at spatial distances of some meters was unexpected because the overall similarities of fungal communities in the soil cores of plot were not significantly different. This is an exciting finding because it suggests that spatial structuring occurs within the root system of an individual. Spatial segregation of different EcM species – mediated by unknown host mechanisms – can ensure colonization by a diverse EcM community on the roots of a given host genotype. Thereby, asymmetric competition between conspecific neighbors can be avoided. We are aware that this suggestion is preliminary because our study includes only few individuals. However, it opens a new avenue to look at spatial structuring of EcM communities.

## Conflict of Interest Statement

The authors declare that the research was conducted in the absence of any commercial or financial relationships that could be construed as a potential conflict of interest.

## Footnotes

^1^http://www.ncbi.nlm.nih.gov/

^2^http://unite.ut.ee

^3^http://chao.stat.nthu.edu.tw
